# Simulation Experiment and Analysis of GNSS/INS/LEO/5G Integrated Navigation Based on Federated Filtering Algorithm

**DOI:** 10.3390/s22020550

**Published:** 2022-01-11

**Authors:** Yuqiang Wang, Bohao Zhao, Wei Zhang, Keman Li

**Affiliations:** 1 Technology and Engineering Center for Space Utilization, Chinese Academy of Sciences, Beijing 100094, China; wangyuqiang19@mails.ucas.ac.cn (Y.W.); likeman21@mails.ucas.ac.cn (K.L.); 2 Key Laboratory of Space Utilization, Chinese Academy of Sciences, Beijing 100094, China; 3 College of Computer Science and Technology, University of Chinese Academy of Sciences, Beijing 100049, China; 4 Aerospace Information Research Institute, Chinese Academy of Sciences, Beijing 100094, China; zhaobh@aircas.ac.cn

**Keywords:** integrated navigation, federated filtering algorithm, GNSS, INS, LEO, 5G, tightly coupled, simulation experiment, NPI

## Abstract

This article examines the positioning effect of integrated navigation after adding an LEO constellation signal source and a 5G ranging signal source in the context of China’s new infrastructure construction. The tightly coupled Kalman federal filters are used as the algorithm framework. Each signal source required for integrated navigation is simulated in this article. At the same time, by limiting the range of the azimuth angle and visible height angle, different experimental scenes are simulated to verify the contribution of the new signal source to the traditional satellite navigation, and the positioning results are analyzed. Finally, the article compares the distribution of different federal filtering information factors and reveals the method of assigning information factors when combining navigation with sensors with different precision. The experimental results show that the addition of LEO constellation and 5G ranging signals improves the positioning accuracy of the original INS/GNSS by an order of magnitude and ensures a high degree of positioning continuity. Moreover, the experiment shows that the federated filtering algorithm can adapt to the combined navigation mode in different scenarios by combining different precision sensors for navigation positioning.

## 1. Introduction

Compared with early navigation methods, modern navigation has entered the information age centered on integrated navigation systems. The integrated navigation system is also called multi-sensor information fusion. According to different task scenarios, using multiple sensors for integrated navigation and optimally fusing multiple types of information according to a certain optimal fusion criterion can be expected to improve positioning accuracy. Commonly used single-sensor navigation methods today include the Inertial Navigation System (Inertial Navigation System, INS), which has autonomous navigation capabilities, but INS positioning error drifts greatly over time [1]. The other one is the Satellite Navigation System (Global Navigation Satellite System, GNSS), which has all-weather, high-precision positioning ability. However, GNSS has the shortcomings of insufficient satellite signal reception and an instantaneous increase in positioning error when the signal is blocked [2]. Integrated navigation is designed based on the complementary performance of a single navigation system; that is, the combination of GNSS/INS can achieve autonomous and high-precision navigation and positioning to a certain extent [3,4]. This combination method has been studied in academia and has been applied in industry. However, in areas such as tall buildings, or tunnels and mines, GNSS signals cannot be received for a long time, and at the same time, the INS positioning error drifts greatly. For example, under the mine, there is no GNSS signal, and it needs to rely on INS for a long time. However, the positioning error of tactical IMU will drift to about 10 m within one minute [5]. In this context, this article proposes to use Low Earth Orbit constellation enhancement and 5G signals as new sensor signal sources for integrated navigation simulation experiments to solve the positioning failure caused by the lack of GNSS signals in specific areas.

Since 2020, the discussion of “new infrastructure” in China has heated up dramatically. As can be seen from relevant research reports [6,7], in the new infrastructure, both satellite internet and 5G infrastructure construction can be used as a National Positioning Infrastructure (NPI).

Satellite internet projects such as Telesat, OneWeb, and Starlink are mainly distributed in the orbit altitude range of 400 km to 1400 km. In China, constellations represented by “Hongyan Constellation”, “Hongyun Project”, “Xingyun Project”, “Celestial Constellations”, and “Milky Way 5G” were designed with heights ranging from 500 km to 1200 km. The above satellite constellations can all be called Low Earth Orbit Satellite Systems (LEO). Nowadays, the accuracy of low-orbit satellite orbit calculation can reach the centimeter-level [8,9,10]. The satellite launch and networking technologies have become more mature so that the number of available satellites has greatly increased, and some technical problems of low-orbit satellite navigation algorithms have been solved. Combining satellite load and application requirements, designing a low-orbit constellation that can be used for navigation, and forming a low-orbit constellation with integrated communication and navigation is an inevitable trend of constellation development [11,12,13]. At the same time, the research of combining LEO and INS for navigation is in the ascendant. Existing research shows that when there is no GNSS signal, the accuracy of integrated navigation using LEO constellation signal and INS is obviously higher than that using INS alone. This proves that using LEO/INS is feasible [14]. Taking advantage of the fast speed of LEO satellite, Doppler frequency shift measurement and positioning technology can be used in LEO satellite. The effect of tight coupling between this technology and INS is also very good, when GNSS is unavailable for 30 s, the final error is reduced from 31.7 m to 8.9 m [15,16,17,18].

As another main component of the new infrastructure, the 5th-generation mobile communication system (5th-generation, 5G) is widely regarded as the foundation of the new generation of the internet. The Internet of Things and Industrial Internet based on 5G have also received widespread attention [19]. The international standards organization 3GPP (3rd Generation Partnership Project), which is leading the 5G communication system protocol standard, has formally defined the commercial application scenarios of 5G positioning requirements in the TR22.872 standard [20]. The use of 5G communication systems for indoor and outdoor positioning is a current research hotspot. Indoor and outdoor positioning algorithms based on the characteristics of 5G millimeter-wave signals have also been extensively studied, and the existing algorithms have reached sub-meter positioning accuracy [21,22,23]. Hybrid positioning schemes based on the fusion of 5G cellular, GNSS/INS are to be studied and developed towards a universal solution for robust positioning of aerial or ground vehicles in urban, rural, and indoor scenarios [24]. Studies have also shown that positioning 5G base stations on both sides of the expressway can improve the robustness and accuracy of the car navigation system on the road [25]. The research of indoor and outdoor joint positioning using 5G shows that when there is no GNSS signal, it is a good choice to use 5G signal instead. Although there have studies use federated filtering, INS is not used as a reference system [26,27]. These studies show that integrated navigation using new sensors needs to be more comprehensive.

In the above context, this article finds that algorithm research and technical application of combined GNSS/INS/LEO/5G navigation and positioning are ascendant in the existing research. Therefore, based on the new sensors included in the new infrastructure, this article proposes implementing a GNSS/INS/LEO/5G integrated navigation simulation experiment by using a federated filtering algorithm to verify the feasibility and positioning accuracy of different integrated navigation positioning schemes.

The integrated navigation simulation verification scheme in this article uses GNSS and LEO satellite constellations simulations to derive the satellite position and speed in the simulation time, which is used as the data source for the integrated navigation satellite positioning. The IMU error model is used to build IMU output specific force and angular velocity models, which are used for integrated navigation, as the INS’s data sources. The 5G ranging signal is simulated by adding random noise based on the given theoretical coordinates of the base station and receiver, based on the 5G ranging signal error model, to provide the ranging value of the 5G signal. Through the implementation of a federated filtering algorithm, the combined navigation and positioning results of the above multi-source signals are output and compared with the true value to verify the navigation and positioning accuracy.

The structure of this article is as follows. The first section is the introduction; the second section introduces the algorithm model used in this article, including algorithm structure frame, simulation scene construction, and simulation method of each signal source; in the third section, the experimental results are compared and explained; finally, in the fourth section, the author puts forward the summary and conclusions of this article. The potential innovations of this article are as follows: simulation verifies the advantages of new integrated navigation using LEO constellation and 5G over traditional integrated navigation; the applicability of the new integrated navigation proposed in this article is presented for the positioning effect in different scenarios. A proposal of information factor allocation using a federated filtering algorithm is proposed when different precision sensors are used.

## 2. The Model for GNSS/INS/LEO/5G Integrated Navigation

At present, the most successful and most applied multi-sensor data fusion method is the federated Kalman filtering method. The federated filter was proposed by Carlson [28]. Because of its flexibility, a small amount of calculation, and good fault tolerance, it has been extensively studied. This method is very flexible for multi-source heterogeneous navigation systems and can support the introduction of new sensor types at any time. This article uses GNSS, INS, LEO, and 5G multi-source heterogeneous navigation information to perform integrated navigation and positioning. There are many types of sensors and rich application scenarios. Therefore, the federated Kalman filter meets the requirements of this article for algorithm functions. The basic algorithm framework of source integrated navigation is the result of this article.

### 2.1. The Structure of Federated Filtering Algorithm

**Figure 1:**
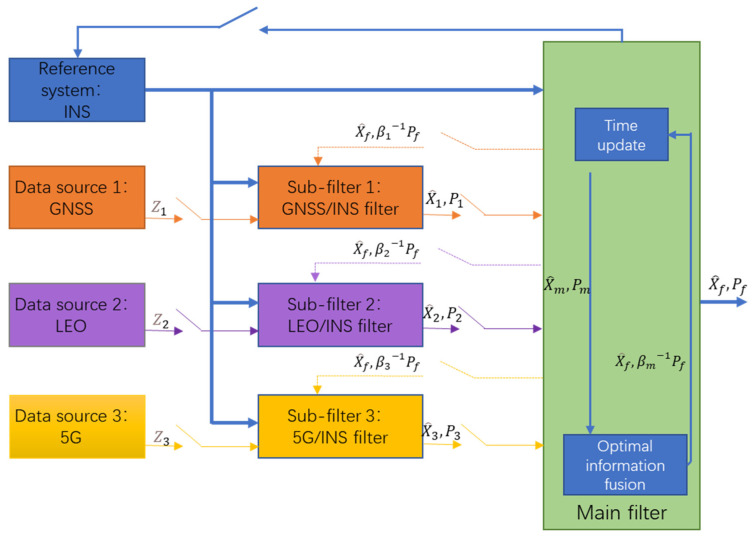
Structure diagram of federated filtering model.

The structure diagram of the federated filter model used in this article is shown in Figure 1.

Each sub-filter of the federated filtering model used in this paper is introduced as follows.

#### 2.1.1. INS Reference System

The reference system is the INS navigation system, whose navigation calculation is mechanically arranged in the ECEF coordinate system. They are:

1. Attitude update:

(1) $$\begin{array}{c} \begin{array}{c} \begin{array}{c} \begin{array}{c} C_{b}^{e}\left(+\right)=\left(\begin{array}{ccc} \cos\omega_{ie}\tau_{i} & \sin\omega_{ie}\tau_{i} & 0\\ -\sin\omega_{ie}\tau_{i} & \cos\omega_{ie}\tau_{i} & 0\\ 0 & 0 & 1 \end{array}\right)C_{b}^{e}\left(-\right)C_{b+}^{b-}\\ \approx C_{b}^{e}\left(-\right)C_{b+}^{b-}-\Omega_{ie}^{e}C_{b}^{e}\left(-\right)\tau_{i} \end{array} \end{array} \end{array} \end{array}$$

where $\omega_{ie}$ is the Earth’s rotational velocity. $\tau_{i}$ is the IMU update interval time. $C_{b}^{e}\left(-\right)$ is the pose matrix for the previous moment which means the transformation is from body coordinate system to ECEF coordinate system. $\Omega_{ie}^{e}$ is the antisymmetric matrix of $\omega_{ie}$. $C_{b+}^{b-}$ is the attitude transfer matrix, calculated using the Rodriguez formula:

(2) $$\begin{array}{c} C_{b+}^{b-}=I_{3}+\frac{\sin\left|\alpha_{ib}^{b}\right|}{\left|\alpha_{ib}^{b}\right|}\left[\alpha_{ib}^{b}\wedge \right]+\frac{1-\cos\left|\alpha_{ib}^{b}\right|}{{\left|\alpha_{ib}^{b}\right|}^{2}}{\left[\alpha_{ib}^{b}\wedge \right]}^{2} \end{array}$$

where $\alpha_{ib}^{b}$ is the rotation vector of the body coordinate system relative to the inertial coordinate system and project in the body coordinate system. $\alpha_{ib}^{b}\wedge$ is the antisymmetric matrix of the rotation vector $\alpha_{ib}^{b}$. The subscripts b+ and superscripts b− in $C_{b+}^{b-}$ means the transformation is from b+ to b−. The + means the current moment. The – means the previous moment.

2. The specific force coordinate transformation:

(3) $$\begin{array}{c} C_{b}^{i}=C_{b}^{i}\left(-\right)C_{b}^{b-},C_{b}^{b-}=I_{3}+\frac{1-\cos\left|\alpha_{ib}^{b}\right|}{{\left|\alpha_{ib}^{b}\right|}^{2}}\left[\alpha_{ib}^{b}\wedge \right]+\frac{1}{{\left|\alpha_{ib}^{b}\right|}^{2}}\left(1-\frac{\sin\left|\alpha_{ib}^{b}\right|}{\left|\alpha_{ib}^{b}\right|}\right){\left[\alpha_{ib}^{b}\wedge \right]}^{2} \end{array}$$

(4) $$\begin{array}{c} f_{ib}^{e}=C_{b}^{e}f_{ib}^{b},C_{b}^{e}=C_{b}^{e}\left(-\right)C_{b}^{b-}-\frac{1}{2}\Omega_{ie}^{e}C_{b}^{e}\left(-\right)\tau_{i} \end{array}$$

where $C_{b}^{i}$ means the transformation is from body coordinate system to inertial coordinate system. $I_{3}$ is the Identity matrix. $f_{ib}^{e}$ is the specific force of the body coordinate system relative to the inertial coordinate system and project in the ECEF coordinate system. $f_{ib}^{b}$ is the specific force of the body coordinate system relative to the inertial coordinate system and project in the body coordinate system. The descriptions of other symbols are mentioned above.

3. Speed and position update:

Speed update:

(5) $$\begin{array}{c} \begin{array}{c} v_{eb}^{e}\left(+\right)\approx v_{eb}^{e}\left(-\right)+\left(f_{ib}^{e}+g_{b}^{e}\left(r_{eb}^{e}\left(-\right)\right)-2\Omega_{ie}^{e}v_{eb}^{e}\left(-\right)\right)\tau_{i}\\ =v_{eb}^{e}\left(-\right)+v_{ib}^{e}+\left(g_{b}^{e}\left(r_{eb}^{e}\left(-\right)\right)-2\Omega_{ie}^{e}v_{eb}^{e}\left(-\right)\right)\tau_{i} \end{array} \end{array}$$

where $v_{eb}^{e}\left(+\right)$ is velocity of the body coordinate system relative to the ECEF coordinate system and project in the ECEF coordinate system in current moment. $g_{b}^{e}$ is the gravity acceleration projected from body system to ECEF system.

Position update:

(6) $$\begin{array}{c} \begin{array}{c} r_{eb}^{e}\left(+\right)=r_{eb}^{e}\left(-\right)+\left(v_{eb}^{e}\left(-\right)+v_{eb}^{e}\left(+\right)\right)\frac{\tau_{i}}{2}\\ \approx r_{eb}^{e}\left(-\right)+v_{eb}^{e}\left(-\right)\tau_{i}+\left(f_{ib}^{e}+g_{b}^{e}\left(r_{eb}^{e}\left(-\right)\right)-2\Omega_{ie}^{e}v_{eb}^{e}\left(-\right)\right)\frac{\tau_{i}^{2}}{2} \end{array} \end{array}$$

where $r_{eb}^{e}\left(+\right)$ is the coordinate of the body coordinate system relative to the ECEF coordinate system and project in the ECEF coordinate system in current moment.

The output of the INS mechanical arrangement solution is position, speed, and attitude, and the calculated results are used as the input of sub-filter and federated filter (the initial value of state estimation). After filtering, the error state quantity of the sub-filter is added to the state estimation for state correction.

#### 2.1.2. GNSS/INS Sub-Filter

**Figure 2:**
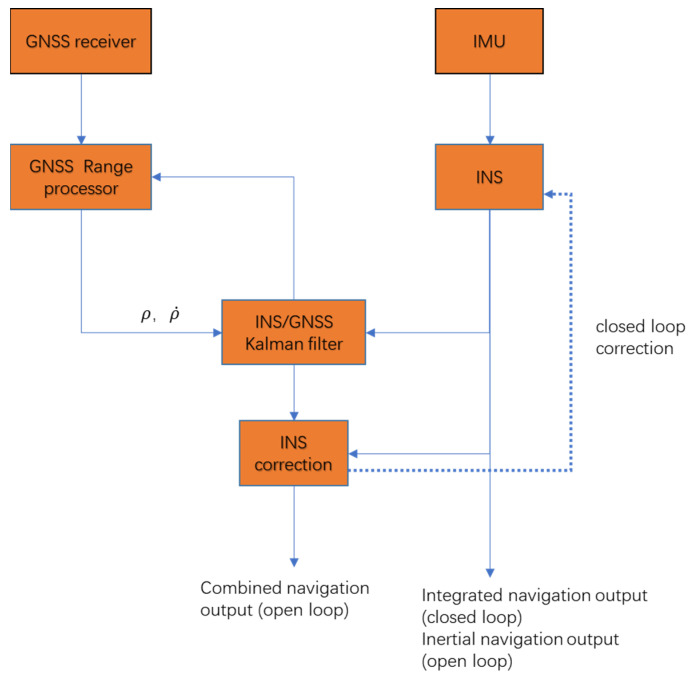
Tightly coupled flow chart of GNSS/INS sub-filter.

The GNSS/INS sub-filter uses tight coupling, and its flow chart is shown in Figure 2 below. In this paper, closed-loop correction is used as the final output of integrated navigation to reduce the error of linearization.

In this paper, the state vector of GNSS/INS sub-filter is based on the error state model, that is:

(7) $$\begin{array}{c} x=\left\{\delta \psi ,\delta v,\delta r,b_{a},b_{g},\delta \rho_{c}^{a},\delta {\dot{\rho }}_{c}^{a}\right\} \end{array}$$

where δψ is the attitude error. δv is the velocity error. δr is the position error. $b_{a}$ is the accelerometer offset. $b_{g}$ is the gyroscope offset. $\delta \rho_{c}^{a}$ is the receiver clock error. $\delta {\dot{\rho }}_{c}^{a}$ is the receiver clock drift.

The transfer matrix of the GNSS/INS sub-filter system is the first-order term:

(8) $$\begin{array}{c} \Phi_{GNSS,\;INS}^{e}\approx \left[\begin{array}{ccccccc} I_{3}-\Omega_{ie}^{e}\tau_{s} & 0_{3} & 0_{3} & 0_{3} & {\hat{C}}_{b}^{e}\tau_{s} & 0 & 0\\ F_{21}^{e}\tau_{s} & I_{3}-2\Omega_{ie}^{e}\tau_{s} & F_{23}^{e}\tau_{s} & {\hat{C}}_{b}^{e}\tau_{s} & 0_{3} & 0 & 0\\ 0_{3} & I_{3}\tau_{s} & I_{3} & 0_{3} & 0_{3} & 0 & 0\\ 0_{3} & 0_{3} & 0_{3} & I_{3} & 0_{3} & 0 & 0\\ 0_{3} & 0_{3} & 0_{3} & 0_{3} & I_{3} & 0 & 0\\ 0 & 0 & 0 & 0 & 0 & 1 & \tau_{s}\\ 0 & 0 & 0 & 0 & 0 & 0 & 1 \end{array}\right] \end{array}$$

where $F_{21}^{e}$ and $F_{23}^{e}$ are:

(9) $$\begin{array}{c} F_{21}^{e}=\left[-\left({\hat{C}}_{b}^{e}{\hat{f}}_{ib}^{b}\right)\wedge \right], \end{array}$$

(10) $$\begin{array}{c} F_{23}^{e}=-\frac{2{\hat{\gamma }}_{ib}^{e}}{r_{eS}^{e}\left({\hat{L}}_{b}\right)}\frac{{\hat{r}}_{eb}^{e^{T}}}{\left|{\hat{r}}_{eb}^{e}\right|} \end{array}$$

where, the ∧ in ${\hat{C}}_{b}^{e}$ means that this is an estimate of the rotation matrix. ${\hat{\gamma }}_{ib}^{e}$ is the gravitational acceleration at the estimated position. $r_{eS}^{e}$ is coordinates of the satellite in ECEF system.

The system covariance matrix is:

(11) $$\begin{array}{c} P\;=\;\left[\begin{array}{ccccccc} SD_{att}{}_{3}^{2} & 0_{3} & 0_{3} & 0_{3} & 0_{3} & 0 & 0\\ 0_{3} & SD_{vel}{}_{3}^{2} & 0_{3} & 0_{3} & 0_{3} & 0 & 0\\ 0_{3} & 0_{3} & SD_{pos}{}_{3}^{2} & 0_{3} & 0_{3} & 0 & 0\\ 0_{3} & 0_{3} & 0_{3} & SD_{ba}{}_{3}^{2} & 0_{3} & 0 & 0\\ 0_{3} & 0_{3} & 0_{3} & 0_{3} & SD_{bg}{}_{3}^{2} & 0 & 0\\ 0 & 0 & 0 & 0 & 0 & SD_{c}^{a}{}^{2} & 0\\ 0 & 0 & 0 & 0 & 0 & 0 & SD_{\mathrm{cd}}^{a}{}^{2} \end{array}\right] \end{array}$$

where, *SD_i_* is the standard deviation of attitude angle, velocity, position, accelerometer deviation, gyroscope deviation, receiver clock error, and receiver clock drift, which is calculated in the form of constant in the simulation.

The innovation matrix is:

(12) $$\delta z_{G,k}^{-}\;=\;\left(\begin{array}{c} \delta z_{\rho ,k}^{-}\\ \delta z_{r,k}^{-} \end{array}\right),\begin{array}{l} \delta z_{\rho ,k}^{-}\;=\;{\left(\;{\tilde{\rho }}_{a,C}^{1}-{\hat{\rho }}_{a,C}^{1-},\;{\tilde{\rho }}_{a,C}^{2}-{\hat{\rho }}_{a,C}^{2-},\cdots \;{\tilde{\rho }}_{a,C}^{m}-\;{\hat{\rho }}_{a,C}^{m-}\right)}_{k}\\ \delta z_{r,k}^{-}\;=\;{\left(\;{\tilde{\dot{\rho }}}_{a,C}^{1}-{\hat{\dot{\rho }\;}}_{a,C}^{1-},\;{\tilde{\dot{\rho }}}_{a,C}^{2}-{\hat{\dot{\rho }\;}}_{a,C}^{2-},\cdots {\tilde{\dot{\rho }}}_{a,C}^{m}-{\hat{\dot{\rho }\;}}_{a,C}^{m-}\right)}_{k} \end{array}$$

where ${\tilde{\rho }}_{a,C}^{1}$, ${\tilde{\dot{\rho }}}_{a,C}^{1}$ is the pseudo-range and pseudo-range rate for measurement $.\;{\hat{\;\rho }}_{a,C}^{1-}$, $\;{\hat{\dot{\rho }}}_{a,C}^{1-}$ is the estimated pseudo-range and pseudo-range rate.

The observation matrix is:

(13) $$\begin{array}{c} H_{G,k}^{\gamma }\approx {\left(\begin{array}{ccccccc} 0_{1,3} & 0_{1,3} & u_{a1}^{\gamma T} & 0_{1,3} & 0_{1,3} & 1 & 0\\ 0_{1,3} & 0_{1,3} & u_{a2}^{\gamma T} & 0_{1,3} & 0_{1,3} & 1 & 0\\ \vdots & \vdots & \vdots & \vdots & \vdots & \vdots & \vdots \\ 0_{1,3} & 0_{1,3} & u_{am}^{\gamma T} & 0_{1,3} & 0_{1,3} & 1 & 0\\ 0_{1,3} & u_{a1}^{\gamma T} & 0_{1,3} & 0_{1,3} & 0_{1,3} & 0 & 1\\ 0_{1,3} & u_{a2}^{\gamma T} & 0_{1,3} & 0_{1,3} & 0_{1,3} & 0 & 1\\ \vdots & \vdots & \vdots & \vdots & \vdots & \vdots & \vdots \\ 0_{1,3} & u_{am}^{\gamma }T & 0_{1,3} & 0_{1,3} & 0_{1,3} & 0 & 1 \end{array}\right)}_{x\;=\;{\hat{x}}_{k}^{-}} \end{array}$$

where $u_{a1}^{\gamma T}$ is the line-of-sight unit vector from the user antenna, a, to satellite, 1.

The observation noise matrix is:

(14) $$\begin{array}{c} R_{G,k}^{\gamma }\approx {\left(\begin{array}{cc} SD_{\rho }{}^{2}*I_{m,m} & 0_{m,m}\\ 0_{m,m} & {\dot{SD_{\rho }}}^{2}*I_{m,m} \end{array}\right)}_{x={\hat{x}}_{k}^{-}} \end{array}$$

The Kalman filtering algorithm is Formulas (15)–(19):

(15) $$\begin{array}{c} {\hat{x}}_{k}^{-}=\Phi_{k-1}{\hat{x}}_{k-1}^{+} \end{array}$$

(16) $$\begin{array}{c} P_{k}^{-}=\Phi_{k-1}\left(P_{k-1}^{+}+\frac{1}{2}Q_{k-1}^{\prime }\right)\Phi_{k-1}^{T}+\frac{1}{2}Q_{k-1}^{\prime } \end{array}$$

(17) $$\begin{array}{c} K_{k}=P_{k}^{-}H_{k}^{T}{\left(H_{k}P_{k}^{-}H_{k}^{T}+R_{k}\right)}^{-1} \end{array}$$

(18) $$\begin{array}{c} \begin{array}{c} {\hat{x}}_{k}^{+}={\hat{x}}_{k}^{-}+K_{k}\left(z_{k}-H_{k}{\hat{x}}_{k}^{-}\right)={\hat{x}}_{k}^{-}+K_{k}\delta z_{k}^{-} \end{array} \end{array}$$

(19) $$\begin{array}{c} P_{k}^{+}=\left(I-K_{k}H_{k}\right)P_{k}^{-} \end{array}$$

where ${\hat{x}}_{k-1}^{+}$ is system state vector in the moment of k−1. $\Phi_{k-1}$ is the system transfer matrix in the moment of k−1. $P_{k-1}^{+}$ is the covariance matrix in the moment of k−1. $Q_{k-1}^{\prime }$ is system noise matrix. $R_{k}$ measurement noise matrix. $K_{k}$ is the Gain matrix. I is the unit matrix.

#### 2.1.3. LEO/INS Sub-Filter

The structure of the LEO/INS sub-filter is similar to that of the GNSS/INS sub-filter. The difference is that the parameters of the LEO constellation model are different from those of the GNSS constellation. The main difference lies in the constellation orbit height, the number of orbital planes, and other parameters. See Section 2.3 for details.

#### 2.1.4. 5G/INS Sub-Filter

The 5G/INS sub-filter adopts a tight coupling structure similar to the GNSS/INS sub-filter. The difference is that the measured value of the 5G/INS sub-filter is the 5G pseudo-range measurement value and pseudo-range rate obtained by adding Gaussian noise to the theoretical distance between the receiver and four 5G base stations. The innovation matrix of the 5G/INS sub-filter is calculated using the measurements derived from INS mechanical arrangement of pseudo-range and pseudo-range rate measurements generated by 5G signals.

#### 2.1.5. Federated Main Filter

The inertial navigation system, as a reference system, is separately combined with GNSS, LEO, and 5G systems to construct a set of local error state Kalman filters, which can be regarded as the first stage of federated filter integrated navigation. In the second stage of federated filter integrated navigation, the output of each sub-filter is combined to form the integrated navigation result. In this paper, the federated main filter is used for information fusion.

The federated master filter is used for information fusion, and the state vector is:

(20) $$\begin{array}{c} {\hat{X}}_{fk}=\left\{\delta \psi ,\delta v,\delta r,b_{a},b_{g},\rho ,\dot{\rho }\right\} \end{array}$$

The relationship between the state vector of the main filter and the sub-filter is:

(21) $$\begin{array}{c} {\hat{X}}_{fk}=P_{fk}\sum_{j=1}^{N}P_{jk}^{-1}{\hat{X}}_{jk} \end{array}$$

where $P_{jk}^{-1}$ is the covariance matrix of sub-filter $j.\;{\hat{X}}_{jk}$ is the state estimate of sub-filter j. N is the number of sub-filter. $P_{fk}$ is the federated filtering covariance matrix, that is:

(22) $$P_{fk}=(\sum_{j=1}^{N}P_{jk}^{-1})$$

The covariance matrix of the federated main filter $P_{fk}$ is obtained by summing the inverse covariance matrices of each sub-filter, and then inverting. Then, find the sum of the product of the state values of each sub-filter ${\hat{X}}_{jk}\;$ and the covariance inverse matrix $P_{jk}^{-1}$. Finally, the final state solution after federated filtering ${\hat{X}}_{fk}\;$ is obtained by multiplying the $P_{fk}$ matrix with the sum $\sum_{j=1}^{N}P_{jk}^{-1}{\hat{X}}_{jk}$. By combining the state values and covariance of each sub-filter, the federated filter value is finally formed according to Formula (21), which is the mathematical significance of federated filtering.

According to the different applications of federated filtering to state vector and covariance matrix, this paper mainly compares the following federated filtering combinations:

1. Federation no reset combination, FNR:

In FNR mode, Formulas (21) and (22) are used as the combination of single point fusion algorithms [29]. When the fusion algorithm is updated, it can be directly used to update the federated integrated navigation output without feedback on the state estimation and covariance of the local filter.

2. Federation fusion reset combination, FFR:

In the FFR mode, the state estimation and error covariance matrix obtained from the information fusion of the federated main filter is fed back to the sub-filter. The sub-filter replaces the corresponding state vector, covariance matrix, and the feedback covariance matrix is multiplied by the coefficient $\beta_{j}$ [28]. $\beta_{j}$ is the information distribution factor of sub-filter j, which satisfies the principle of information fusion; that is, for N sub-filters, there are $\sum_{j=1}^{N}\beta_{j}=1$.

3. Federation Zero reset combination, FZR:

In FZR mode, after the state estimation result of the sub-filter is input to the main filter, the state values of all the sub-filters are set to zero, and the corresponding covariance matrix element values are set to the initial value [29].

### 2.2. Simulation Scene Construction

**Figure 3:**
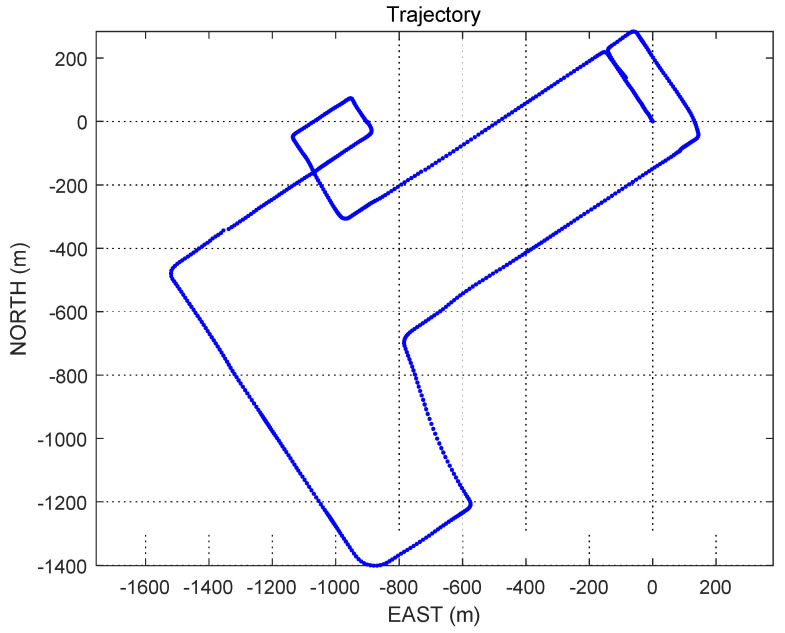
Trajectory diagram of simulation experiment.

The motion truth file in this article refers to the data provided by the open-source project “UrbanNavDataset” [30]. The data set was collected in a typical urban canyon in Tokyo on 19 December 2018, and its trajectory in the northeast geodetic coordinate system is shown in Figure 3.

The experimental scene in this paper is vehicle navigation at a low speed on the ground (in the ECEF coordinate system, the speed of each axis is less than that of 20 m/s), the site is the urban high-rise canyon area, and the carrier vehicle has multiple right-angle turning maneuvers. At the same time, this paper simulates and constructs different experimental scenes by setting the range of mask angle and azimuth angle.

The parameter patterns of each simulation scene in this paper are shown in Table 1.

**Figure 4:**
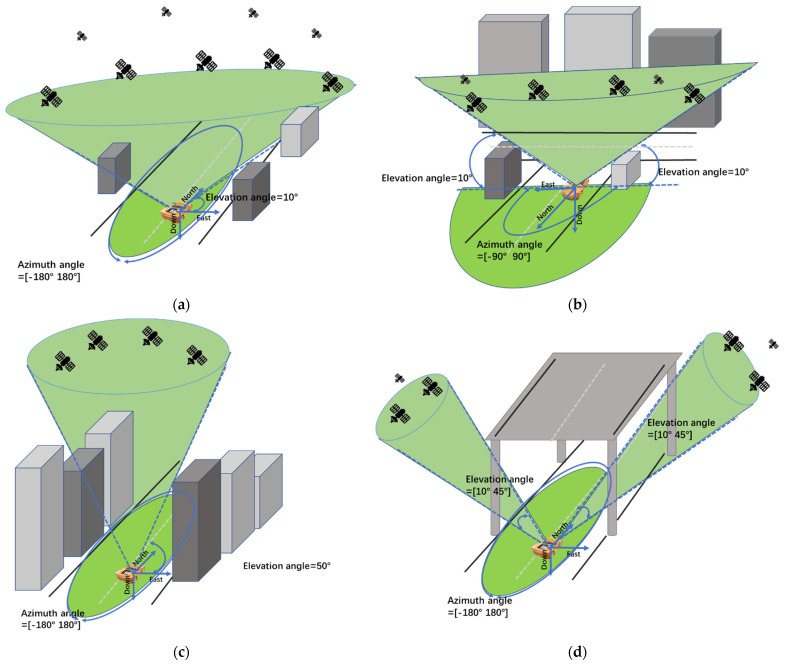
Schematic diagram of simulation scene (**a**) Open scene; (**b**) Semi-occluded scene; (**c**) Surrounding occluded scene; (**d**) Headspace occluded scene.

The schematic diagram of each simulation scene is shown in Figure 4.

It can be seen from Table 1 and Figure 4 that the simulation scene in this paper includes four modes: Open scene, Semi-occluded scene, Surrounding occluded scene, and Headspace occluded scene. In the Open scene (Figure 4a), there is no restriction on azimuth angle, the mask angle is low so that there are more visible satellite data. The azimuth of the Semi-occluded scene (Figure 4b) ranges from −90° to 90°, significantly reducing the number of visible satellites. For the Surrounding occluded scene (Figure 4c), the mask angle is raised to 50°, and the azimuth angle is not restricted to simulate the urban canyon environment. As a result, the number of visible satellites in Surrounding occluded scene is also small. In the Headspace occluded scene (Figure 4d), the mask angle is limited to 10° to 45°, and the azimuth angle is not limited to simulate the environment under the urban viaduct. Naturally, the number of visible satellites in Headspace occluded scene is lower.

### 2.3. Simulation Method of Each Signal Source

#### 2.3.1. GNSS and LEO Constellation Simulation

Both GNSS and LEO constellation simulations in this paper are Walker constellations and aim to optimize constellation coverage with a given number of satellites. Walker constellation is proposed after Ballard’s improvement in literature [31], forming a constellation system widely used in the field of orbit design [32].

The parameters of the Walker constellation include the number of satellites *T*, the number of orbits *P*, and the number of satellites per orbit *S*. All *P* orbital planes have the same orbital inclination *i* (relative to the equatorial plane). On each orbital plane, *S* satellites are evenly distributed on the orbital plane with an angular distance of 360°/*S*. The ascending points of each orbital plane are evenly distributed on the equatorial plane with an interval of 360°/*P*. In order to maintain the relative position relationship between satellites of different orbital planes, the strategy of the equal time interval between satellites of adjacent orbital planes passing through their ascending intersection is adopted. Therefore, the relative phase must be an integer multiple of 360°/*T*, *F* can be any integer between 0 and P−1. By giving three parameters *T*, *P*, *S*, and orbital inclination *i*, the constellation form can be completely determined, namely the Walker constellation.

Table 2 shows the parameters of the GNSS/LEO Walker constellation simulation. The constellation distribution operation diagram after STK software simulation is shown in the following figure.

**Figure 5:**
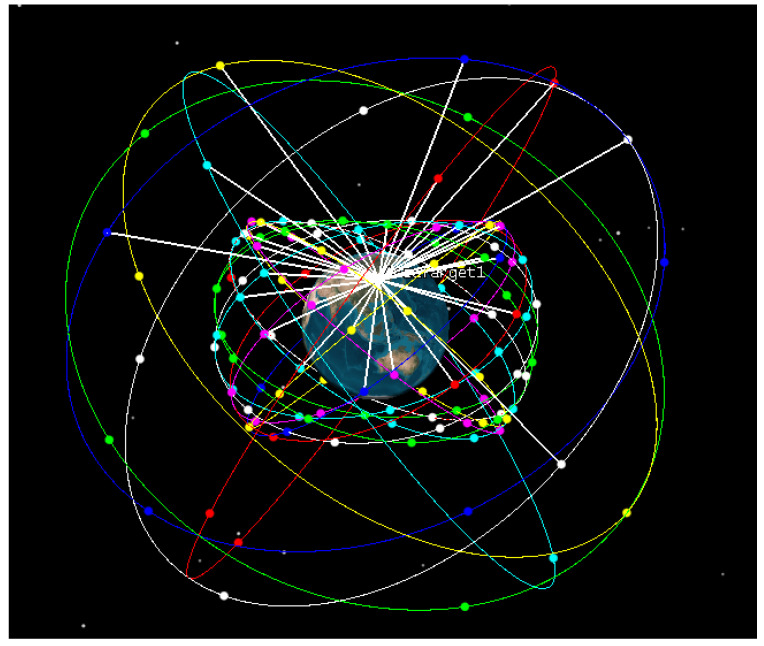
GNSS_LEO constellation STK simulation results.

The simulation results after GNSS and LEO were simulated and connected with the ground are shown in Figure 5.

**Figure 6:**
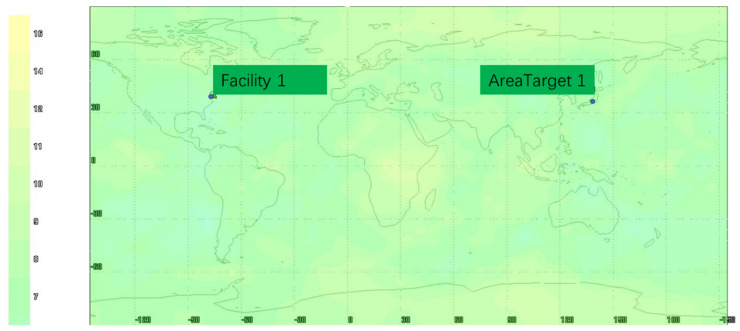
Global visibility analysis of GNSS constellation.

**Figure 7:**
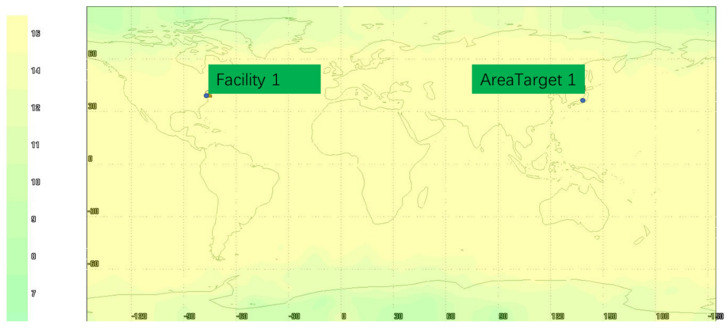
Global visibility analysis of LEO constellation.

**Figure 8:**
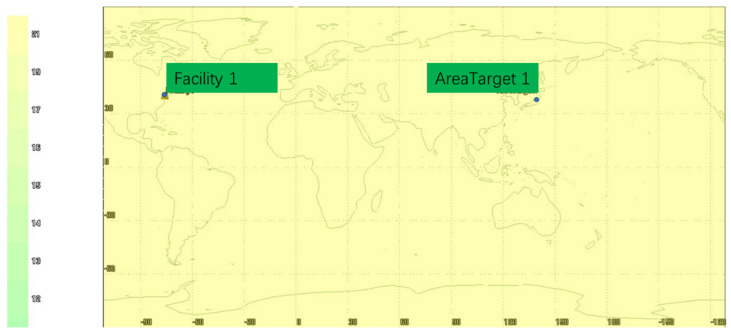
Global visibility analysis of GNSS+LEO constellation.

The visibility analysis is shown in Figure 6, Figure 7 and Figure 8. As can be seen from Figure 6, Figure 7 and Figure 8, the selected target area is 139.776° E~139.802° E and 35.569° N~35.687° N, and the visibility of the target area is 100% within 24 h of simulation. According to the global visibility analysis report of the GNSS+LEO constellation in Figure 8, it can be concluded that the GNSS and LEO constellation simulated in this paper can provide satellite-ranging signal data source for integrated navigation in the target area.

Based on the GNSS/LEO constellation simulation to obtain the satellite position and speed, the pseudo-range measurement value of satellite navigation can be obtained using Formula (23) below:

(23) $$\begin{array}{c} \hat{\rho }=\rho +\delta \rho_{I}+\delta \rho_{T}+\delta \rho_{SIS}+\delta \rho_{ct}+\delta \rho_{sc}+\delta \rho_{c}+\delta {\dot{\rho }}_{cd}\times ∆t \end{array}$$

where $\hat{\rho }$ is the measured value of satellite navigation pseudo-range. ρ is the theoretical distance between satellite position and receiver. $\delta \rho_{I}$ is the ionospheric error. $\delta \rho_{T}$ is tropospheric error. $\delta \rho_{SIS}$ is the spatial signal propagation error, which is mainly caused by multipath, non-line-of-sight, and diffraction phenomena. $\delta \rho_{ct}$ is code tracking error. $\delta \rho_{sc}$ is the clock error of satellite. $\delta \rho_{c}$ is the clock error of receiver. $\delta {\dot{\rho }}_{cd}$ is the receiver clock drift. ∆t is the time interval for satellite navigation.

The measurement value of pseudo distance ratio is obtained by Formula (24):

(24) $$\begin{array}{c} \hat{\dot{\rho }}=\dot{\rho }+\delta {\dot{\rho }}_{cd}+\delta {\dot{\rho }}_{ct} \end{array}$$

where $\hat{\dot{\rho }}$ is the measurement of satellite navigation pseudo-range ratio. $\dot{\rho }$ is the theoretical distance. $\delta {\dot{\rho }}_{cd}$ is the receiver clock drift. $\delta {\dot{\rho }}_{ct}$ is the range-rate tracking error.

The error parameters used for satellite navigation in this paper are given in Table 3.

Table 3 shows related parameters used by GNSS and LEO satellite navigation models. On the basis of theoretical distance and range rate obtained by simulation, range error and range rate error are added, which are the measurement values of pseudo-range and pseudo-range rate of GNSS and LEO satellite navigation models.

#### 2.3.2. IMU Model Simulation

The IMU error model in this paper is:

(25) $$\begin{array}{c} \begin{array}{c} {\tilde{f}}_{ib}^{b}=b_{a}+\left(I_{3}+M_{a}\right)f_{ib}^{b}+w_{a},\\ \;{\tilde{\omega }}_{ib}^{b}=b_{g}+\left(I_{3}+M_{g}\right)\omega_{ib}^{b}+G_{g}f_{ib}^{b}+w_{g} \end{array} \end{array}$$

where ${\tilde{f}}_{ib}^{b}$ is the output specific force of IMU. $f_{ib}^{b}$ is the theoretical value of specific force. $b_{a}$ is the acceleration deviation. $M_{a}$ is the scale factor and cross-coupling error of the accelerometer. $w_{a}$ is the random noise of the accelerometer. $b_{g}$ is the gyroscope bias. ${\tilde{\omega }}_{ib}^{b}$ is the IMU output angular rate. $\omega_{ib}^{b}$ is the theoretical value of angular velocity. $M_{g}$ is the gyroscope scale factor and cross-coupling error. $G_{g}$ is the acceleration of gravity-related gyro bias. $w_{g}$ is the gyroscope random noise.

Table 4 shows the simulation parameters of the IMU model. In this paper, INS navigation calculation is carried out under the ECEF coordinates system, and the IMU error model value (Table 4) is added based on the theoretical value of specific force $f_{ib}^{b}$ and theoretical value of angular velocity $\omega_{ib}^{b}$ obtained by simulation, which is the measured value of IMU.

#### 2.3.3. 5G Signal Simulation

The simulation of 5G ranging signal in this paper, obtained from Formulas (26) and (27), is obtained by adding simulation signal error based on theoretical distance value and range rate between the base station and receiver, namely:

(26) $$\begin{array}{c} {\hat{\rho }}_{5G}=\rho_{5G}+\delta \rho_{c-5G}+\delta {\dot{\rho }}_{cd-5G}\times ∆t \end{array}$$

(27) $$\begin{array}{c} {\hat{\dot{\rho }\;}}_{5G}={\dot{\rho }}_{5G}+\delta {\dot{\rho }}_{cd-5G}+\delta {\dot{\rho }}_{cd-5G} \end{array}$$

where ${\hat{\rho }}_{5G}$ and ${\hat{\dot{\rho }}}_{5G}$ are the pseudo-range measurement and pseudo-range rate measurement of the 5G signal, respectively. $\rho_{5G}$ and ${\dot{\rho ˙}}_{5G}$ are the theoretical distance and the distance rate. $\delta \rho_{c-5G}\;$ is the clock bias of the receiver. $\delta {\dot{\rho }}_{cd-5G}$ is the clock drift deviation. ${\dot{\rho }}_{ct-5G}$ is the distance rate tracking error. ∆t is a 5G positioning time interval.

Table 5 shows the parameters of the 5G-ranging models used in this paper. In order to simulate the coverage of 5G signal on the motion track, in this paper, according to the coordinates of the center point of the motion track, taking the base station coverage radius r as the axis, four base stations are evenly distributed in the same height plane with an azimuth interval of 90°.The coordinates of each base station are calculated by the above distribution method and 5G model parameters.

## 3. Experimental Results

### 3.1. Comparison of Location Results in Different Scenarios

After setting parameters in different scenarios, the results of the simulation experiment are as follows. Note that FNR mode is adopted for federated filtering in this section.

#### 3.1.1. Open Scene

The positioning result after setting the azimuth angle and mask angle of the open scene in Section 2.2 is shown in the figures below.

**Figure 9:**
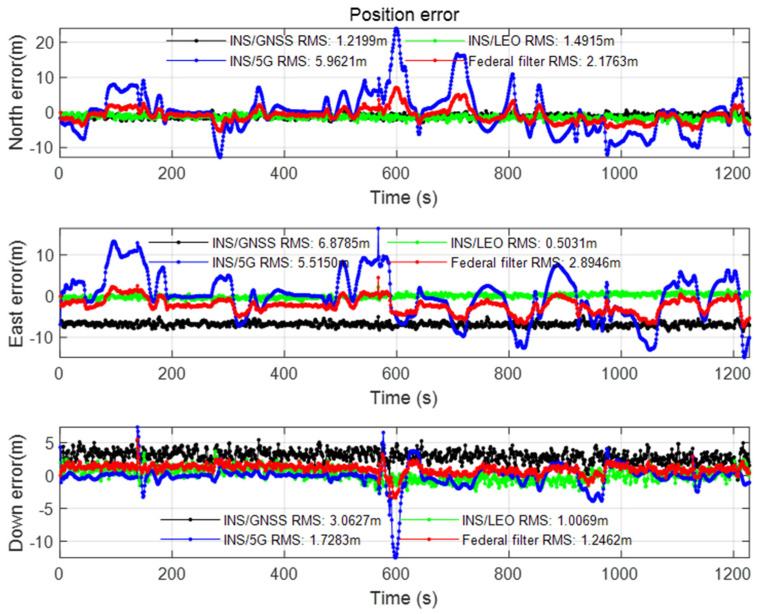
Position error of the Open scene.

**Figure 10:**
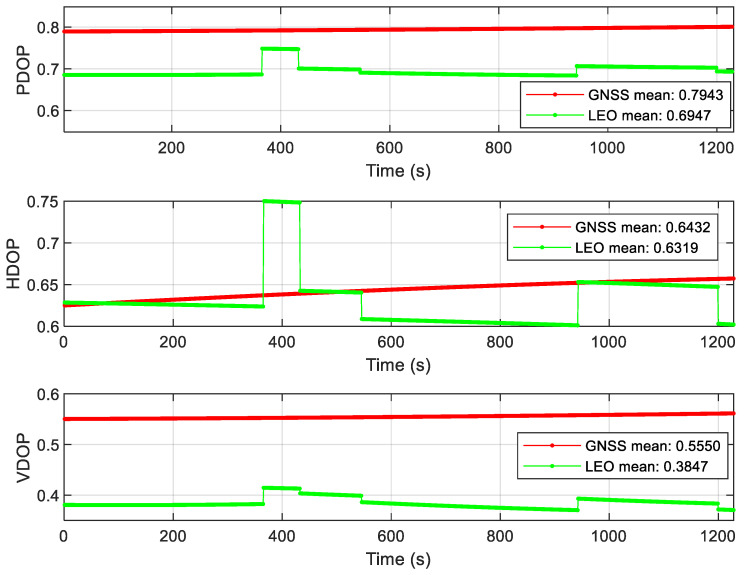
DOP value of the Open scene.

**Figure A1:**
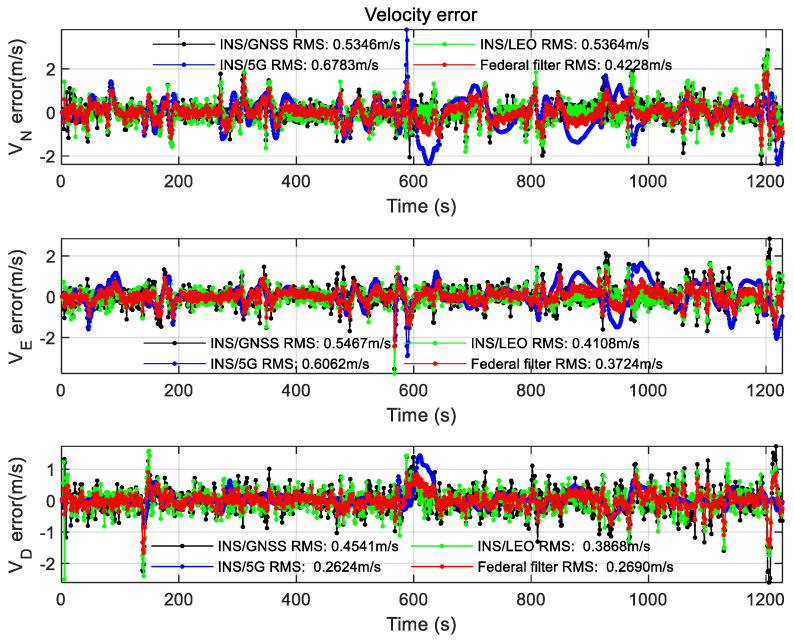
Velocity error of the Open scene.

**Figure A2:**
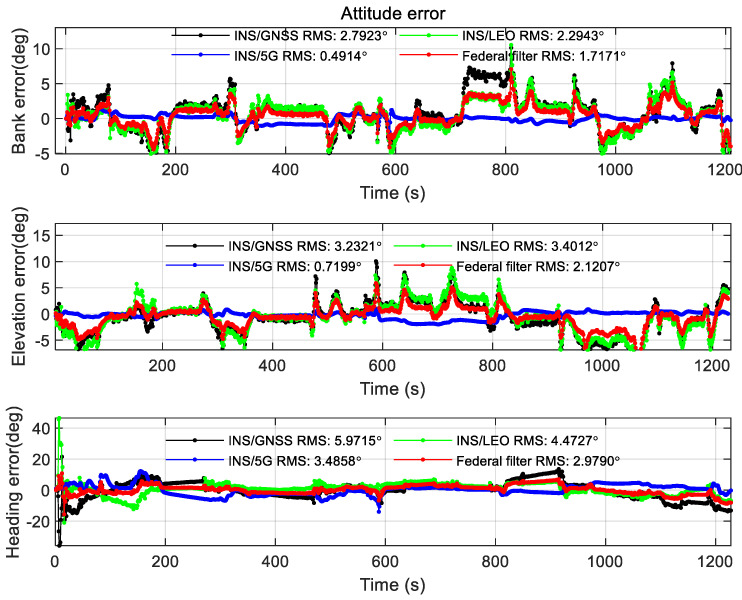
Attitude error of the Open scene.

As can be seen from Figure 9, after the LEO constellation was introduced in the open scene, the LEO/INS position accuracy increased by an order of magnitude to sub-meter in the east direction compared with GNSS/INS position accuracy. Affected by the poor accuracy of 5G/INS position, the overall federated filtering accuracy is in meter level, but it is higher than the accuracy of single 5G/INS position, and the positioning error is more stable than 5G/INS, the positioning convergence speed is faster, and the positioning continuity is more guaranteed. According to the DOP value analysis of GNSS and LEO constellations in Figure 10, it can be seen that the geometric distribution of the LEO constellation is significantly improved compared with that of the GNSS constellation. The results of velocity error and attitude error of the Open scene can be seen from Figure A1 and Figure A2 in Appendix A. The error values after using the federated filtering algorithm in this article both are relatively stable.

#### 3.1.2. Semi-Occluded Scene

The positioning result after setting the azimuth angle and mask angle of the semi-occlusion scene in Section 2.2 is shown in the figure below.

**Figure 11:**
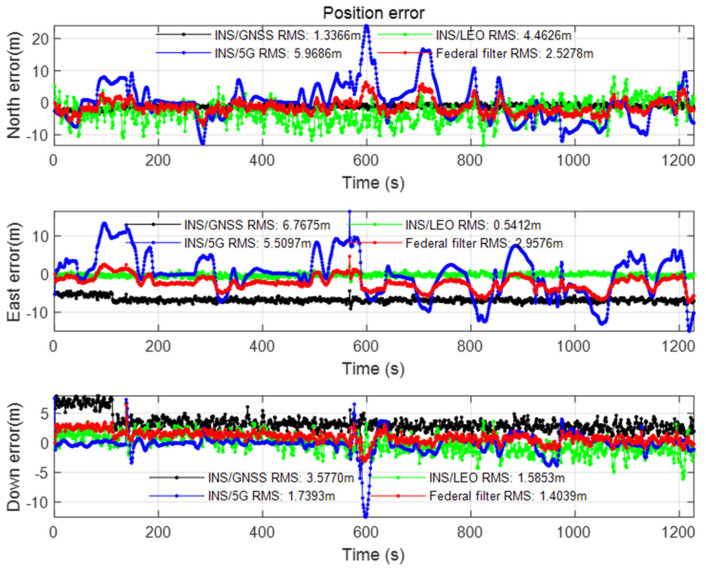
Position error of the Semi-occluded scene.

**Figure 12:**
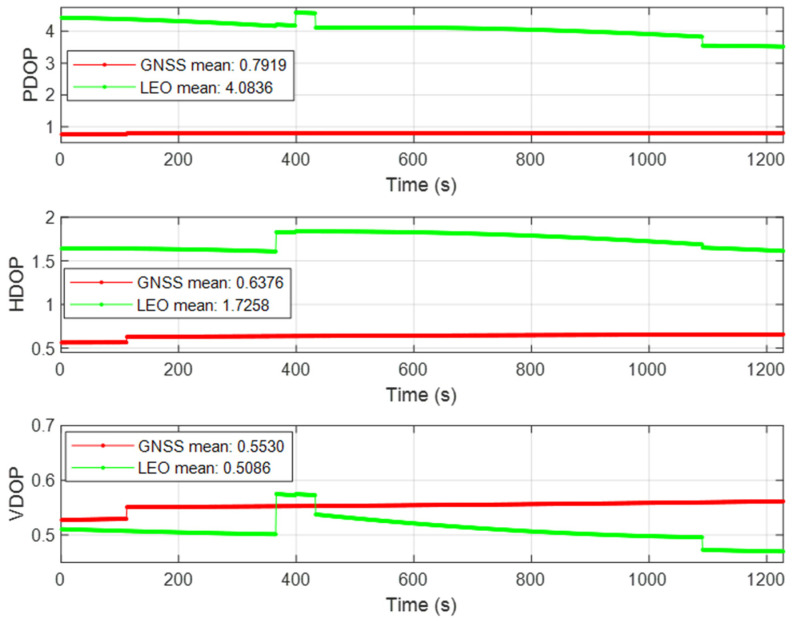
DOP value of the Semi-occluded scene.

**Figure A3:**
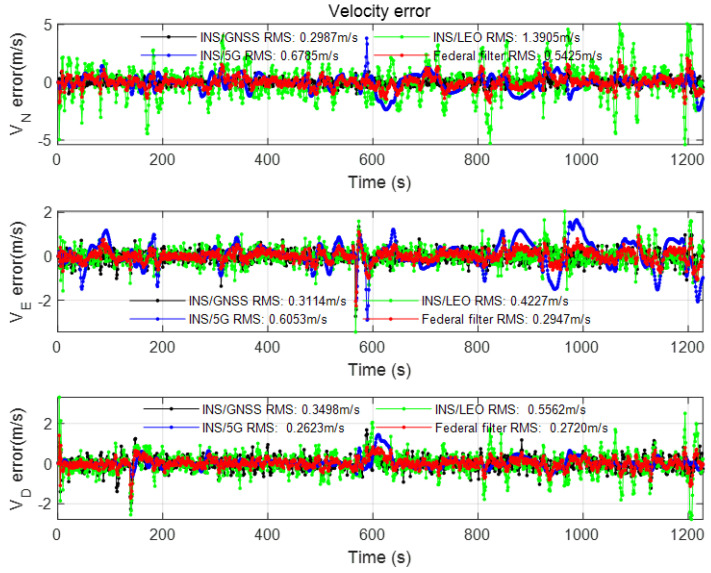
Velocity error of the Semi-occluded scene.

**Figure A4:**
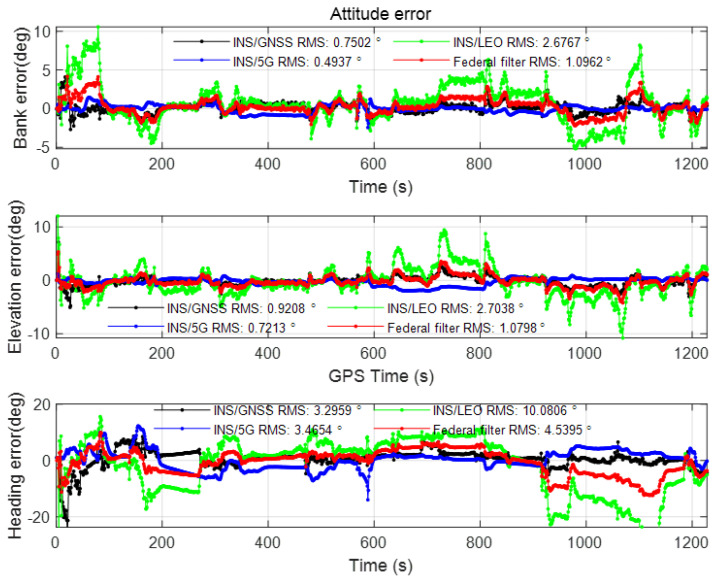
Attitude error of the Semi-occluded scene.

It can be seen from Figure 11 that in the Semi-occluded scene, because the azimuth range is reduced by half compared with the Open scene, the positioning accuracy of LEO/INS has obvious influence, and the position error in the north direction is significantly larger than that of GNSS/INS. It can be seen from Figure 12 that the geometric configuration of the LEO constellation in the Semi-occluded scene is obviously worse than that of GNSS in three-dimensional position and horizontal position, which further affects the positioning accuracy of LEO. Due to the influence of high orbit height, the positioning accuracy in the semi-occluded scene has little influence on the GNSS constellation. Due to the better fault tolerance of the federated filtering algorithm and the visible feature of the 5G base station, the 5G ranging data used in this paper is not occluded, so 5G/INS positioning is used as a supplement, which makes the positioning accuracy of the whole federated filtering about 2 m. The results of velocity error and attitude error of the Semi-occluded scene can be seen from Figure A3 and Figure A4 in Appendix A. The fluctuation of velocity error and attitude error of the Semi-occluded is larger than the value in the Open scene.

#### 3.1.3. Surrounding Occluded Scene

The positioning results are shown in the figure below after setting the azimuth and mask angle of the Surrounding occluded scene in Section 2.2.

**Figure 13:**
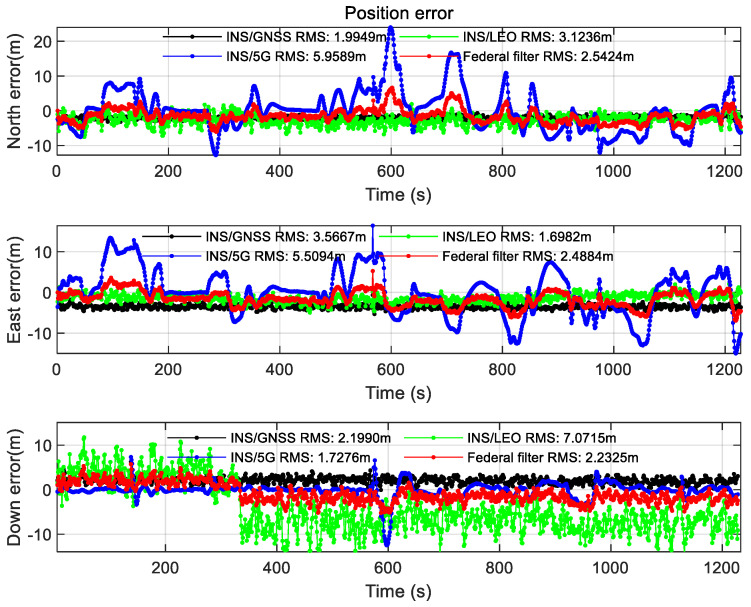
Position error of the Surrounding occluded scene.

**Figure 14:**
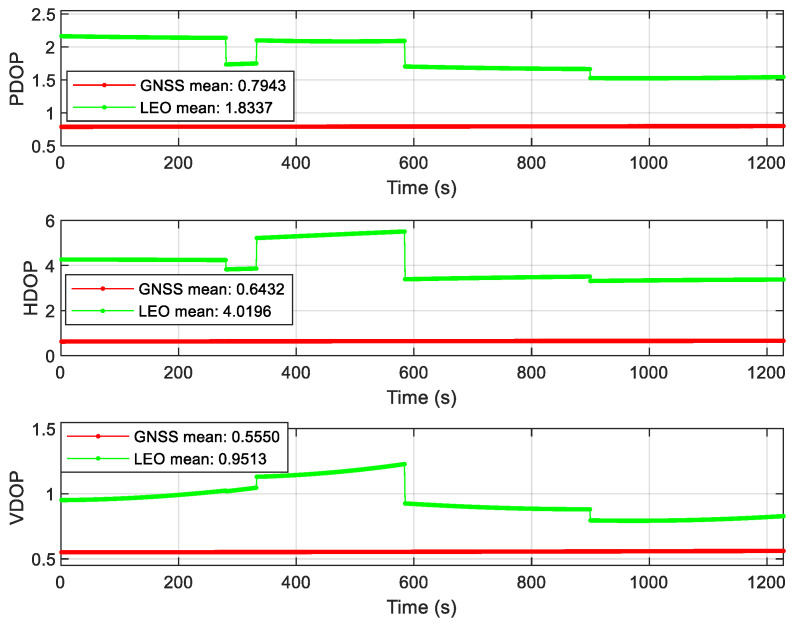
DOP value of the Surrounding occluded scene.

**Figure A5:**
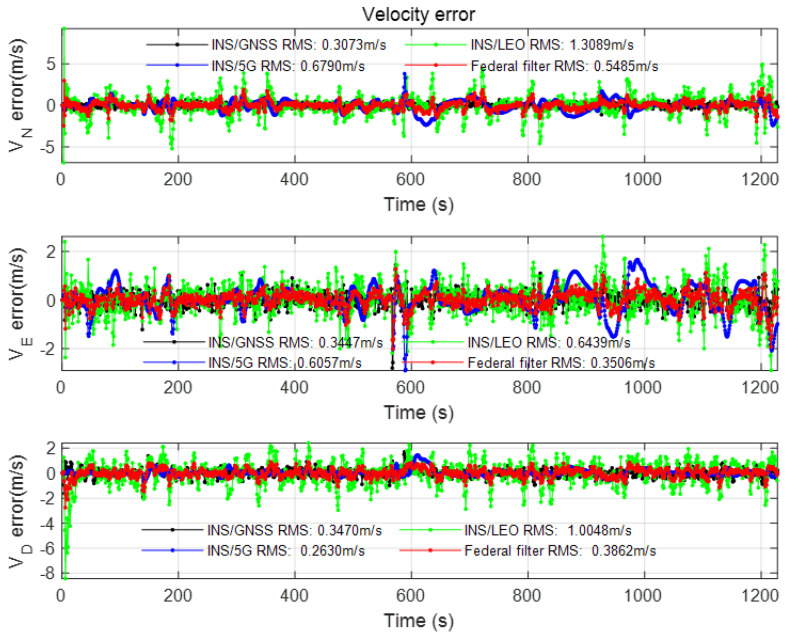
Velocity error of the Surrounding occluded scene.

**Figure A6:**
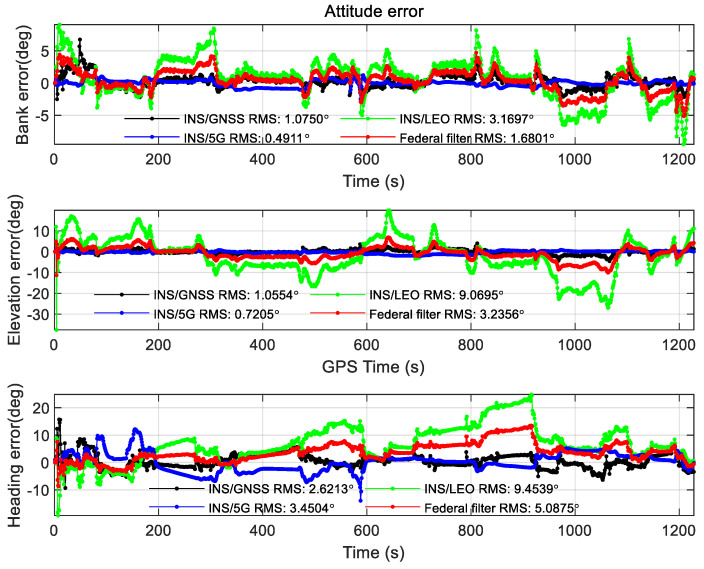
Attitude error of the Surrounding occluded scene.

As can be seen from Figure 13, under the influence of the increase of mask angle, the LEO/INS positioning accuracy becomes worse, but the accuracy after GNSS/INS filtering is improved. The overall accuracy after federated filtering is about 2 m. As can be seen in Figure 14, in the Semi-occlusion scenario, the geometric configuration of the LEO constellation is greatly limited due to the high mask angle limit. The results of velocity error and attitude error of the Surrounding occluded scene can be seen from Figure A5 and Figure A6 in Appendix A. The error value of the sub-filter will be reduced after using federated filtering.

#### 3.1.4. Headspace Occluded Scene

The positioning result after setting the azimuth angle and mask angle of the headspace occluded scene in Section 2.2 is shown in the figure below.

**Figure 15:**
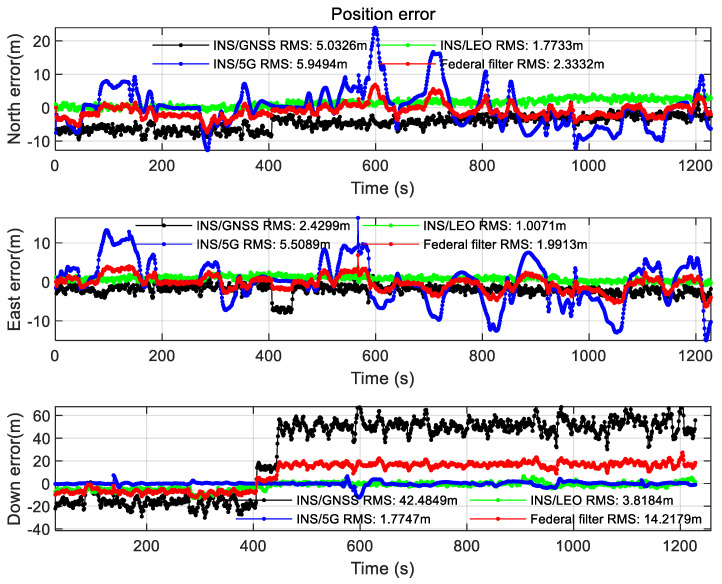
Position error of the Headspace occluded scene.

**Figure 16:**
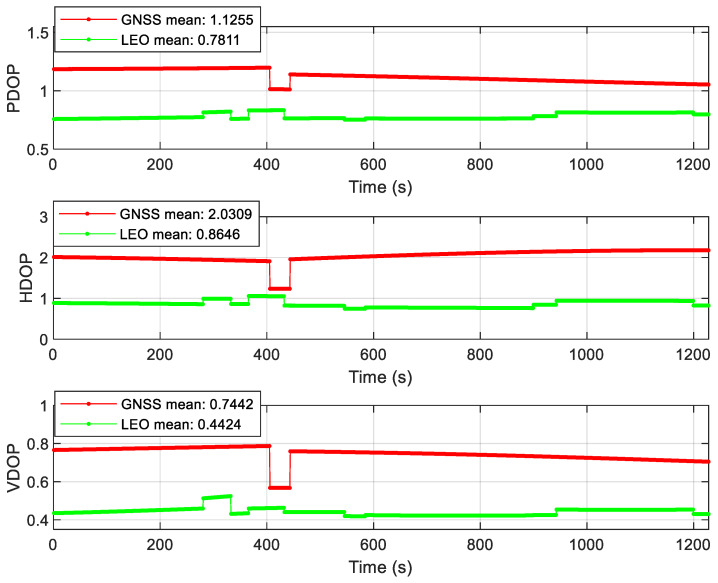
DOP value of the Headspace occluded scene.

**Figure A7:**
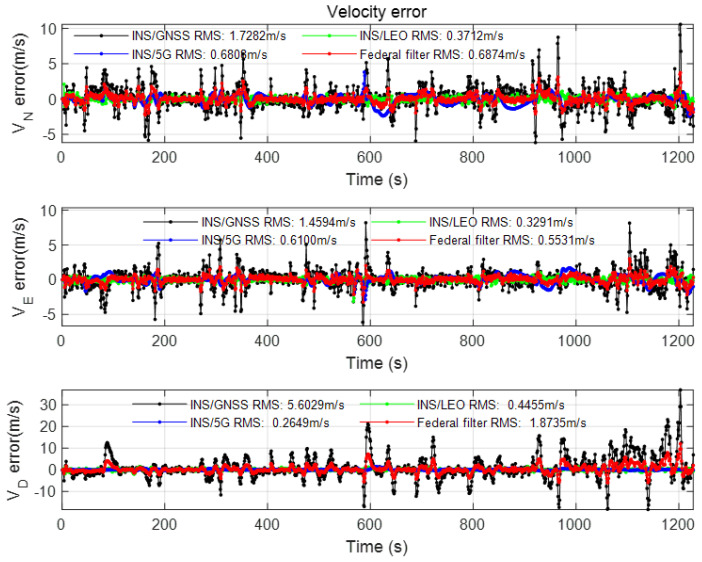
Velocity error of the Headspace occluded scene.

**Figure A8:**
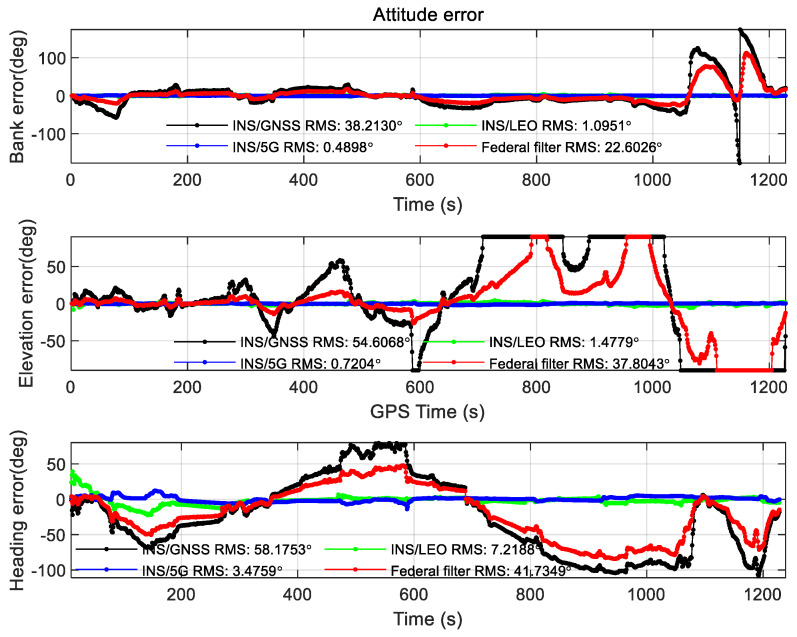
Attitude error of the Headspace occluded scene.

As can be seen from Figure 15, under the Headspace occluded scenario, the GNSS constellation is limited by the range of mask angle, so the GNSS/INS positioning error increases by one order of magnitude in the celestial direction. The LEO constellation geometry has an obvious advantage in this scene, so the positioning error is not affected. Due to the geometric configuration advantage of the LEO constellation, the positioning accuracy of the federated filter is higher than that of GNSS/INS in this scene. As can be seen from Figure 16, the DOP value of the GNSS constellation in the Headspace occlusion scenario is significantly higher than that of the LEO constellation. The results of velocity error of the Headspace occluded scene can be seen from Figure A7 in Appendix A. The velocity error of INS/GNSS positioning has obvious fluctuation, and the fluctuation of attitude error has exceeded the acceptable range. The attitude angle error increases by one order of magnitude which can be seen from Figure A8 in Appendix A.

It can be seen from the positioning results in Section 3.1, the positioning errors and constellation DOP values are quite different in different scenarios. The positioning results of the federated filtering algorithm in FNR mode are compared in the following table.

In Table 6, this subsection compares the positioning results of four scenes. Experiments show that GNSS/INS, LEO/INS, and 5G/INS alone have poor positioning accuracy in a certain scene. However, by using the federated filtering algorithm, the positioning accuracy can be stabilized. At the same time, by comparing the positioning results in different scenarios, it can be found that the convergence effect of the federated filtering positioning results after the introduction of LEO constellation and 5G signals is more stable and faster than GNSS/INS. Plus, the positioning continuity is better guaranteed. In particular, the LEO/INS positioning effect is better in the Headspace occlusion scene.

### 3.2. Comparison of Different Information Factor Allocation and Location Results of Different Modes for Federated Filtering

After comparing the positioning accuracy of integrated navigation in different scenes according to the different performance of positioning accuracy of GNSS, LEO, and 5G signals, we conclude that different sensors can be set with different information factors. So, the next experimental comparison can be made to verify the influence of information factor allocation of federated filtering in the same mode on positioning accuracy. Therefore, in the Open scene, the experimental results of different information factors under the FZR mode of federated filtering are compared, as shown in the following figures.

**Figure 17:**
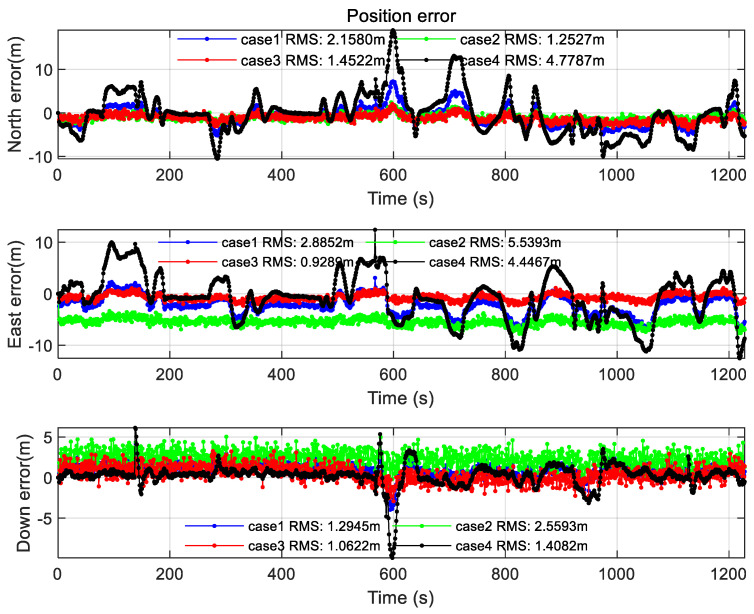
Federated filtering position errors of different information factors.

**Figure A9:**
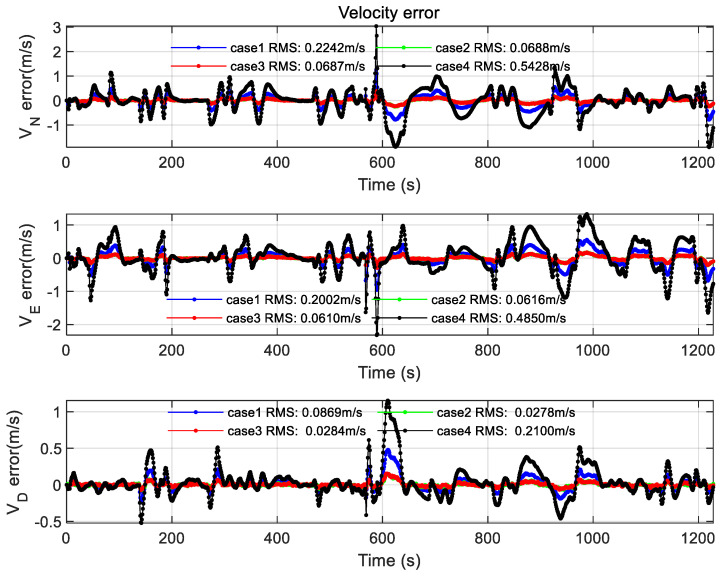
Federated filtering velocity errors of different information factors.

**Figure A10:**
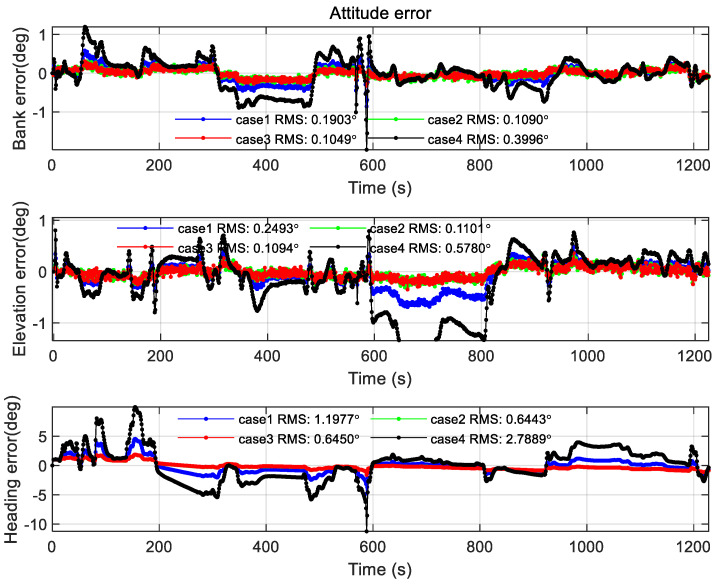
Federated filtering attitude errors of different information factors.

In Figure 17, the position error of different information factors is shown. The velocity error and attitude error can be seen from Figure A9 and Figure A10 in Appendix A. The values of different information factors can be seen in the following table.

As shown in Table 7, if a sub-filter positioning accuracy is higher than several orders of magnitude than another sub-filter, the information factor ratio of this sub-filter needs to be close to 1. The above conclusion can be seen from the compared simulation results. In this way, the positioning results of federated filter can be significantly improved. It can be seen from Figure 17, since the LEO/INS positioning accuracy is higher than GNSS/INS and 5G/INS, Case3 can improve the overall positioning accuracy by increasing the proportion of the LEO/INS information factor to 80%.

At the same time, this paper also compares the federated filtering positioning accuracy of different modes under the same scale factor (using the percentage in Case1), as shown in the figure below.

**Figure 18:**
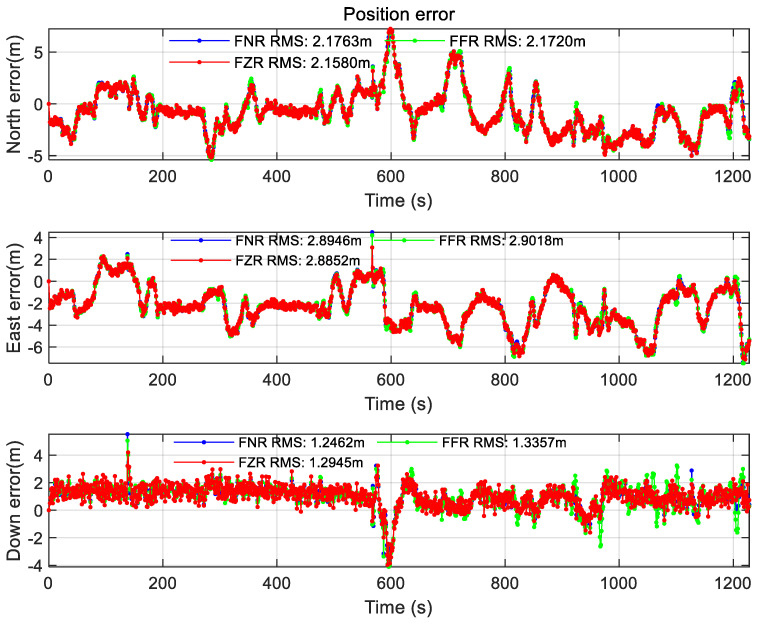
Position error of different mode federated filtering.

**Figure 19:**
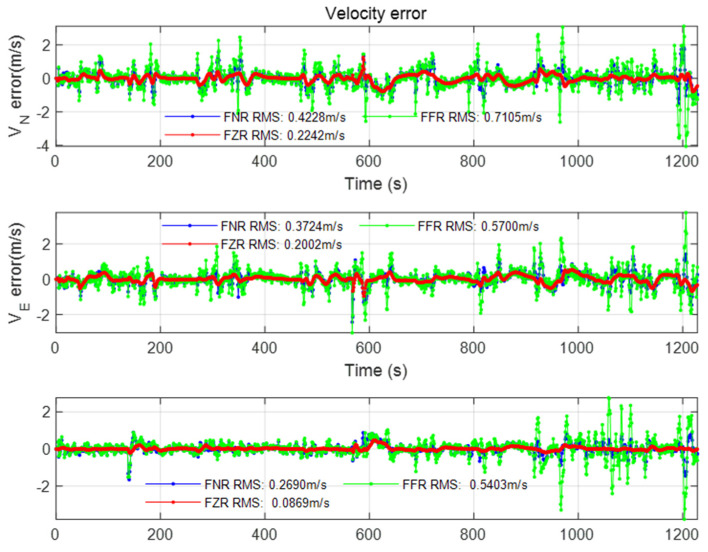
Velocity error of different mode federated filtering.

**Figure 20:**
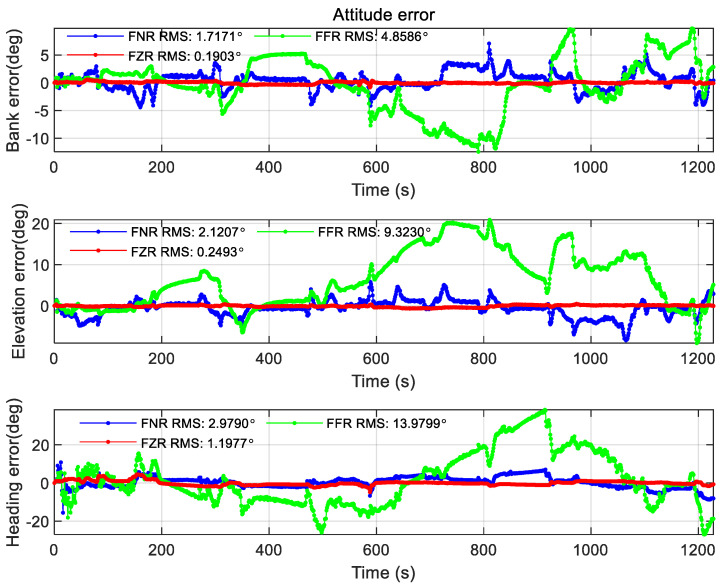
Attitude error of different mode federated filtering.

From the comparison of Figure 18, Figure 19 and Figure 20, it is found that the federated filter under different modes has no obvious difference in position error. However, the FFR mode performs poorly in speed error and attitude angle error, which is reflected in the large error fluctuations, and the attitude angle error reaches 10° in magnitude. After analysis, it is considered that the filter in this paper adopts the error state vector model, and different modes have different ways to deal with the covariance matrix. The covariance matrix of FFR mode feedback leads to the instability of the sub-filter, which leads to the speed error increasing to the level of 1 m/s after 100 s and the attitude angle error increasing to the level of 10° after 200 s. FNR mode does not affect the sub-filter, so the positioning result has no influence. The velocity error in FNR mode is less than 0.5 m/s, and the attitude angle error is less than 2.5°. The FZR mode resets the covariance matrix of the sub-filter, so the error of velocity and attitude angle does not change much. The velocity error is less than 0.3 m/s and the attitude angle error is less than 1.2° in the FZR mode.

In this subsection, the positioning results under different federated filtering modes are compared, and it is found in the experiment that FZR mode and FNR mode have less influence on the sub-filter covariance matrix than FFR mode, and the positioning accuracy is better.

In Section 3, by comparing positioning results in different scenarios, positioning results in different information factors, and positioning results in different federated filtering modes, it is found that higher information factors should be set for sensors with higher accuracy according to different application scenarios. If the error state vector modeling is adopted, the FNR and FZR mode positioning results of federated filtering are better.

## 4. Conclusions

In this work, INS, GNSS, LEO, and 5G signal sources were simulated, and integrated navigation simulation experiments were carried out using tightly coupled and federated filtering algorithms. By setting the azimuth angle and the satellite visible altitude angle, the positioning results in four occlusion scenes, namely, Open scene, Semi-occluded scene, Surrounding occluded scene, and Headspace occluded scene, are compared. The main conclusions that the paper can support are as follows: (1) the experimental results show that, after adding the LEO constellation, the geometric configuration of the LEO constellation can significantly improve the accuracy factor, which provides strong support for the positioning effect of the Headspace occlusion scene, and improve the positioning accuracy of the original INS/GNSS by an order of magnitude; (2) after the addition of the 5G ranging signal, due to the uninterrupted characteristics of the 5G ranging signal, the overall positioning continuity of federated filtering was greatly improved; (3) by allocating different scale factors, the experiments show that the federated filtering algorithm can combine sensors with different precision for navigation and positioning, to adapt to the integrated navigation modes in different scenes, and open up a new idea for new sensor integrated navigation.

The future improvement of this paper lies in that this paper only tests the single point positioning mode with new LEO and 5G sensors based on INS/GNSS. In the future, we can study higher-precision positioning algorithms, including but not limited to RTK positioning and PPP positioning. The data used in LEO navigation and positioning in this paper is the satellite position and velocity obtained by simulating the satellite constellation, and there is no measured data source for verification. The simulation of 5G ranging signal only adopts pseudo-range measurement simulation with noise added based on theoretical value. There is no contrast experiment with the way of positioning by specifying positioning protocol in communication standard. The difference of positioning accuracy between the two ways has not been specified yet.

## Figures and Tables

**Table 1:** Simulation scene parameters.

Category	Mask Angle (deg)	Azimuth Range (deg)
Open scene	10	[−180, 180]
Semi-occluded scene	10	[−90, 90]
Surrounding occluded scene	45	[−180, 180]
Headspace occluded scene	[10, 45]	[−180, 180]

**Table 2:** GNSS/LEO Walker constellation parameters.

Parameters	GNSS	LEO
Number of satellite—*T*	30	120
Number of orbits—*P*	6	12
Number of satellites per orbit—*S*	5	10
Number of relative phase multiple—*F*	1	1
Angle of orbital inclination—*i*	55 deg	40 deg
Altitude of orbit—*h*	20,183.65 km	1139.9 km

**Table 3:** GNSS/LEO navigation model parameters.

Parameters	Value	
	GNSS	LEO
Satellite positioning time interval (s)— ∆t	1	1
Mask Angle (deg, it can be adjusted according to different scenarios)	10	10
Zenith ionosphere error SD (m)— $\delta \rho_{I}$	2	1.5
Zenith troposphere error SD (m)— ${\delta \rho }_{T}$	0.2	0.15
Space signal error SD (m)— $\delta \rho_{SIS}$	1	1
Code tracking error SD (m)— $\delta \rho_{ct}$	0.5	1
Satellite clock error SD(m)— $\delta \rho_{sc}$	0.5	1
Distance rate tracking error SD (m/s)— $\delta {\dot{\rho }}_{ct}$	0.02	0.05
Initial estimated position of receiver (m, in ECEF coordinate system)	[0,0,0]	[0,0,0]
Receiver clock offset (m)— $\delta \rho_{c}$	10,000	10,000
Receiver clock drift (m/s)— $\delta {\dot{\rho }}_{cd}$	100	100

**Table 4:** IMU model parameters.

Parameters	Value
Accelerometer bias(m/s^2^)—$b_{a}$	[0.0088, −0.0127, 0.0078]
gyroscope bias (deg/h)—$b_{g}$	[10, 10, 10]
Accelerometer scale factor and cross-coupling error— $M_{a}$	$\left[\begin{array}{ccc} 500 & -300 & 200\\ -150 & -600 & 250\\ -250 & 100 & 450 \end{array}\right]\times 10^{-6}$
Gyroscope scale factor and cross-coupling error— $M_{g}$	$\left[\begin{array}{ccc} 400 & -300 & 250\\ 0 & -300 & -150\\ 0 & 0 & -350 \end{array}\right]\times 10^{-6}$
Gyroscope gravity acceleration correlation bias (deg/h/g)—$G_{g}$	$\left[\begin{array}{ccc} 0.9 & -1.1 & -0.6\\ -0.5 & 1.9 & -1.6\\ 0.3 & 1.1 & -1.3 \end{array}\right]$
Accelerometer random noise PSD root(m·s^−1.5^)$—w_{a}$	$9.80665\times 10^{-4}$
Gyro random noise PSD root(rad·s^−0.5^)—$w_{g}$	$2.9089\times 10^{-6}$

**Table 5:** 5G navigation model parameters.

Parameters	Value
Time interval (s)—∆t	1
Receiver clock offset (m)— $\delta \rho_{c-5G}$	10,000
Receiver Clock drift (m/s)— $\delta {\dot{\rho }}_{cd-5G}$	100
The number of base stations	4
Base station signal coverage radius (m)— r	400
Signal number tracking error (m)— $\delta \rho_{ct-5G}$	0.2
Range rate tracking error (m/s)— $\delta {\dot{\rho }}_{ct-5G}$	0.1

**Table 6:** Positioning results.

Category		Positioning Results							
		Open Scene		Semi-Occluded Scene		Surrounding Occluded Scene		Headspace Occluded Scene	
Position error RMS (m)	North	2.1763	mean (m) 2.1057	2.5278	mean (m) 2.2964	2.5424	mean (m) 2.4211	2.3332	mean (m) 2.8475
	East	2.8946		2.9576		2.4884		1.9913	
	Down	1.2462		1.4039		2.2325		4.2179	
Velocity error RMS (m/s)	North	0.4228	mean (m/s) 0.3541	0.5425	mean (m/s) 0.3697	0.5485	mean (m/s) 0.4284	0.6874	mean (m/s) 1.0380
	East	0.3724		0.2947		0.3506		0.5531	
	Down	0.2670		0.2720		0.3862		1.8735	
Attitude error RMS (deg)	North	1.7171	mean (deg) 2.2723	1.0962	mean (deg) 2.2385	1.6801	mean (m) 3.3344	22.6026	mean (deg) 34.0473
	East	2.1207		1.0798		3.2356		37.8043	
	Down	2.9790		4.5395		5.0875		41.7349	
GNSS	PDOP	0.7943		0.7919		0.7943		1.1255	
	HDOP	0.6432		0.6376		0.6432		2.0309	
	VDOP	1.0798		1.0731		1.0798		4.4642	
LEO	PDOP	0.6947		4.0836		1.8337		0.1811	
	HDOP	0.6319		1.7258		4.0196		0.8646	
	VDOP	0.9153		1.7811		4.5272		1.8796	

**Table 7:** GNSS/INS, LEO/INS, and 5G/INS sub-filter information factor ratio.

Category	The Percentage (%)		
	GNSS	LEO	5G
Case1	33	34	33
Case2	80	10	10
Case3	10	80	10
Case4	10	10	80

## Data Availability

Not applicable.

## Appendix A

In order to avoid reading difficulties caused by too many figures, this article puts the results of velocity error and attitude error in Appendix A.

As can be seen from Figure A1 and Figure A2, the velocity error and attitude error of the Open scene is shown. The error values after using the federated filtering algorithm in this article both are relatively stable.

It can be seen from Figure A3 and Figure A4, the fluctuation of velocity error and attitude error of the Semi-occluded is larger than the value in the Open scene, which shows that the influence of simulation scene block on velocity and attitude error begins to play a role.

As can be seen from Figure A5 and Figure A6, in the Surrounding occluded scene, LEO constellation is obviously limited, and the error of velocity and attitude is large. However, the error value of the sub-filter will be reduced after using federated filtering.

It can be seen from Figure A7 and Figure A8, with the decrease of visibility of GNSS constellation, the velocity error of INS/GNSS positioning has obvious fluctuation, and the fluctuation of attitude error has exceeded the acceptable range.

It can be seen from Figure A9 and Figure A10, different information factors have different effects on the velocity error and attitude error. The information factor values in different case can be seen from Table 7.
